# Evaluation of the therapeutic efficacy of a MEK inhibitor (TAK-733) using ^18^F-fluorodeoxyglucose-positron emission tomography in the human lung xenograft model A549

**DOI:** 10.1007/s12149-015-0984-4

**Published:** 2015-05-27

**Authors:** Seigo Ishino, Hiroshi Miyake, Patrick Vincent, Ikuo Mori

**Affiliations:** Integrated Technology Research Laboratories, Pharmaceutical Research Division, Takeda Pharmaceutical Company Limited, 26-1, Muraoka-Higashi 2 chome, Fujisawa, Kanagawa 251-8555 Japan; Drug Discovery Unit, Pharmaceutical Research Division, Takeda Pharmaceutical Company Limited, Fujisawa, Kanagawa 251-8555 Japan; Discovery Biology, Takeda San Diego, San Diego, CA 9212 USA; Research and Development Consulting, Encinitas, CA 92024 USA; Former Employee of Takeda San Diego, San Diego, CA 9212 USA

**Keywords:** TAK-733, ^18^F-FDG-PET, MEK inhibitor, A549 xenograft rat

## Abstract

**Objective:**

The aim of this study was to evaluate the potential of ^18^F-fluorodeoxyglucose-positron emission tomography (^18^F-FDG-PET) for monitoring the therapeutic efficacy of TAK-733, an inhibitor of mitogen-activated protein kinase kinase, in nude rats bearing A549 (human lung carcinoma) xenografts.

**Methods:**

TAK-733 was administered orally by gavage to nude xenograft rats for 2 weeks, at dosage levels of 0 (0.5 % w/v methylcellulose solution), 1, 3, and 10 mg/kg/day (*n* = 8/dose). Tumor size was measured before treatment (day 0), and on days 1, 3, 7, 9, 11, and 14. PET scans were performed pretreatment (day 0), and on days 2, 4, 7, 10, and 14. Tracer accumulations in tumor tissue were quantified as the mean standard uptake value (SUVmean).

**Results:**

No deaths or treatment-related body weight losses occurred during the study period. TAK-733 showed dose-dependent inhibition of tumor growth and ^18^F-FDG uptake in tumor tissue. At a dosage of 10 mg/kg, TAK-733 treatment produced a statistically significant reduction in tumor weight from day 11 compared with the vehicle group (*P* < 0.05). Tumor growth was inhibited in the 10 mg/kg group with a treated/control value of 31 % on day 14. The SUVmean on day 2 in this dosage group was statistically lower than that observed on day 0, and that seen in the vehicle group on day 2 (*P* < 0.05 for both comparisons). Furthermore, this reduction in SUVmean at 10 mg/kg was maintained over time. In the two lower dosage groups (1 and 3 mg/kg), SUVmean gradually increased over time.

**Conclusions:**

^18^F-FDG-PET enabled early determination of late anti-tumor activity in response to TAK-733 treatment.

## Introduction

Mitogen-activated protein kinase kinases 1 and 2 (MEK1/2) are tyrosine and serine/threonine dual-specific kinases, included in the mitogen-activated protein kinase (MAPK) cascade, which play an essential role in cell proliferation, cell cycle regulation, survival, and migration [1, 2]. Inappropriate MAPK pathway activation is observed in ≥50 % of human cancers, including colon, lung, breast, pancreas, melanoma, ovary, and kidney cancer [3]. TAK-733 is a novel, potent, selective, non-adenosine triphosphate (ATP)-competitive, allosteric inhibitor of MEK1/2 kinase, which has the potential to inhibit cancer proliferation [4].

Positron emission tomography (PET) is widely used in clinical cancer diagnosis and is increasingly being used to assess the response to anti-cancer therapy. ^18^F-fluorodeoxyglucose (^18^F-FDG) is a glucose analog that is taken up into tumor cells by glucose transporters. Within cells, it is phosphorylated by hexokinase to ^18^F-FDG phosphate, which becomes trapped inside the cells; unlike glucose 6-phosphate, ^18^F-FDG phosphate is not a substrate for further glycolytic metabolism, and its level of dephosphorylation to ^18^F-FDG is low [5]. Most tumors express large numbers of glucose transporters together with high hexokinase activity, and therefore exhibit high ^18^F-FDG uptake; so the primary clinical application of PET uses ^18^F-FDG as a biomarker to detect changes in glucose metabolism. Effective therapies that kill tumor cells and/or target cell glucose metabolism would be expected to reduce local glucose utilization relative to pretreatment values, although certain therapies can cause transient increases in glucose metabolism too.

^18^F-FDG-PET imaging has proved itself a valuable tool in the successful clinical development of several oncology drugs, for example Pfizer’s sunitinib for the treatment of gastrointestinal stromal tumors and advanced kidney cancer, and Novartis’s imatinib for the treatment of gastrointestinal stromal tumors [6, 7]. In clinical studies of these agents, ^18^F-FDG-PET imaging has revealed significant metabolic reduction in tumor tissue prior to tumor burden reduction and/or clinical benefit. This ability to detect an early indication of drug efficacy has provided confidence for the further development of these drugs, especially with regard to continued investment decisions.

Prior to many clinical studies, in vivo preclinical studies are often performed to characterize the drug of interest. Drug activity in animal studies is commonly determined by measuring post-treatment changes in the size of tumors that have been implanted subcutaneously. However, the accuracy of the measurement is affected by the thickness of the subcutaneous fat layer, as well as by hair and fur. Recently, ^18^F-FDG-PET has been used with high-resolution, small animal scanning equipment, as an adjunct to traditional measures of anti-tumor activity, to evaluate metabolic responses in these xenograft models [6].

This study examined the efficacy of TAK-733 against A549 human, non-small cell lung carcinoma (NSCLC) xenografts in nude rats. ^18^F-FDG-PET assessment of therapeutic efficacy was compared with tumor growth inhibition [treated/control (*T*/*C*)], measured by conventional caliper assessment of tumor size. The broad aims of the study were to evaluate: (1) whether ^18^F-FDG uptake can be used as an early indicator of tumor growth inhibition by TAK-733; and (2) whether the PET response correlates with anti-tumor activity.

## Materials and methods

### TAK-733

**Fig. 1:**
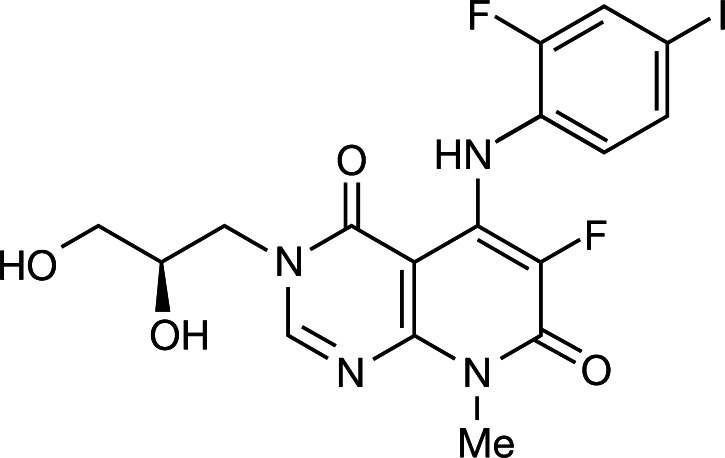
Chemical structure of TAK-733

TAK-733, 3-[(2R)-2,3-Dihydroxypropyl]-6-fluoro-5-[(2-fluoro-4-iodophenyl)amino]-8-methylpyrido [2,3-d]pyrimidine-4,7(3H,8H)-dione (Takeda California, CA, USA) was prepared in 0.5 % w/v methylcellulose 400 (Wako Chemical USA, Richmond, VA, USA) (Fig. 1) [4].

### Animals and husbandry

All studies were conducted at Molecular Imaging, Inc., Ann Arbor, MI, USA. The rats were housed and maintained in accordance with Institutional Animal Care and Use Committee (IACUC), state, and federal guidelines. The 32 animals were 7–9 weeks old at the time of tumor implantation, and were fed irradiated Rodent Diet 5053 (LabDiet™) and water ad libitum. They were housed in static cages with Bed-O’Cobs™ bedding inside Biobubble^®^ Clean Rooms that provided HEPA-filtered air into the bubble environment at 100 complete air changes per hour. All treatments and body weight determinations were carried out in the bubble environment. The temperature was maintained at 22 ± 2 °C with a humidity range of 30–70 %.

Test animals were implanted subcutaneously to the right flank with 30–60 mg tumor fragments of A549 human, non-small cell lung carcinoma (NSCLC) using a 10-gauge trocar needle. All animals were observed for clinical signs at least once daily. Animals with tumors in excess of 5 g, or with ulcerated tumors, were euthanized, as were those found in obvious distress or in a moribund condition.

### Treatment

Treatment began when the mean estimated tumor weight for all groups (vehicle, 1, 3, 10 mg/kg, *n* = 8/group) was 250 mg (range of group means 214–297 mg). All animals weighed ≥145 g at the initiation of therapy. Mean group body weights at first treatment were well matched (range of group means 167–190 g). All animals were dosed according to individual body weight on the day of treatment (10 mL/kg) as indicated in the protocol.

### PET imaging

PET was performed using a Siemens R4 small animal µPET scanner and ^18^F-FDG radiotracer (IBA Molecular). Pretreatment PET scans were acquired on day 0. Post-treatment imaging occurred on days 2, 4, 7, 10, and 14. The animals were fasted for at least 6 h before the start of imaging to minimize blood glucose levels. They were subsequently anesthetized with 1.75 % isoflurane in air and injected in the tail vein with approximately 400 µCi of ^18^F-FDG. ^18^F-FDG uptake occurred under anesthesia for 1.5 h. Body temperature was maintained with a thermostat-regulated, re-circulating water-heated pad and measured as rectal temperature. Individual body weights were recorded before each imaging session. Static emission data were acquired for 13 min using a single bed position.

### PET image analysis and endpoints

The PET list mode data were converted to 2-dimensional (2D) sinograms, corrected for random coincidences, and normalized for scanner uniformity. Attenuation-corrected PET images were reconstructed using an iterative 2D, ordered subsets expectation maximization (OSEM) algorithm. The entire tumor was segmented in the PET images using the Amira Segmentation Editor. Tumor regions of interest (ROIs) were drawn in three orthogonal planes by a well-trained technician according to caliper-based tumor length and width, and an ellipsoidal tumor volume was then interpolated by the software. Software-calculated tumor volumes were compared against caliper-based volumes to ensure consistency (average difference 1.08 %). The mean PET tumor signal was calculated from all voxels within the ROIs and converted to ^18^F activity units. The mean ^18^F-FDG standardized uptake value (SUVmean) was calculated according to the relation:

$$ {\text{SUVmean}} = {\text{mean radioactivity in the tumor }}(\mu {\text{Ci}}/{\text{g}}) \times {\text{body weight }}\left( {\text{g}} \right)/{\text{injected dose }}\left( {{\text{decay}} - {\text{corrected}}} \right) \, (\mu{\text{Ci}}). $$

### Measurements and endpoints

Testing was carried out by adhering to the general principles established by the groups of Schabel, Skipper, Griswold, Corbett, Leopold, Ross, and the National Cancer Institute (NCI) [8–11]. Body weights and tumor size were measured before treatment (day 0), and on days 1, 3, 7, 9, 11, and 14. Tumor weight (mg) was estimated from caliper measurements using the formula for the volume of a prolate ellipsoid assuming unit density:

$$ {\text{Tumor weight }}\left( {\text{mg}} \right) = \left( {L \times W^{2} } \right)/2, $$

where *L* and *W* are the respective orthogonal tumor length and width measurements (mm).

The primary endpoint used to evaluate efficacy was %*T*/*C*, which was defined as the ratio of median tumor weight in the treated versus control (vehicle) group × 100. In this experiment, %*T*/*C* was evaluated on each day. The NCI standard for significant anti-tumor activity in xenograft models is *T*/*C* ≤ 41 % [12–15]. All animals were observed for clinical signs at least once daily.

### Histology

After the last PET measurement, animals were killed and subcutaneous tumors were extracted. Formalin-fixed (10 %), paraffin-embedded sections (4 μm) of resected specimens from tumor tissue were taken for staining. Hematoxylin and eosin (H&E) staining was conducted according to standard protocols. Ki-67 immunohistochemical staining was performed using the labeled streptavidin biotinylated antibody (LSAB) method with an autostaining system (Ventana Medical Systems Inc., USA) according to the manufacturer’s protocol. MIB-1 antibody (DAKO), a monoclonal murine antibody specific for human nuclear antigen Ki-67, was used as the primary antibody in a 1:100 dilution.

The Ki-67 index (%) was estimated by counting the percentage of Ki-67-positive cell nuclei per 200–300 tumor cells in the region of the tumor with the greatest density of staining, which in most instances corresponded to areas with the highest mitotic activity, as determined by H&E staining.

### Statistics

Differences in tumor volumes between pairs of treatment groups were assessed on days 0, 1, 3, 7, 9, 11, and 14. Estimated tumor weights were compared by applying a pairwise Wilcoxon rank-sum test, with *P* value (*P* < 0.05) adjustment by the method of Holm (non-parametric test).

The same statistical methodology was used in the analysis of the ^18^F-FDG-PET data to: (1) compare normalized ^18^F-FDG SUVmean between groups at each imaging time point; and (2) compare normalized SUVmean between imaging time points for each group.

The Ki-67 index was analyzed statistically using the Shirley–Williams test at a two-tailed significance level of 0.025, assuming a dose-related trend was performed. The Shirley–Williams test was performed using the SAS function PROBMC.

## Results

### Anti-tumor effects of TAK-733

**Fig. 2:**
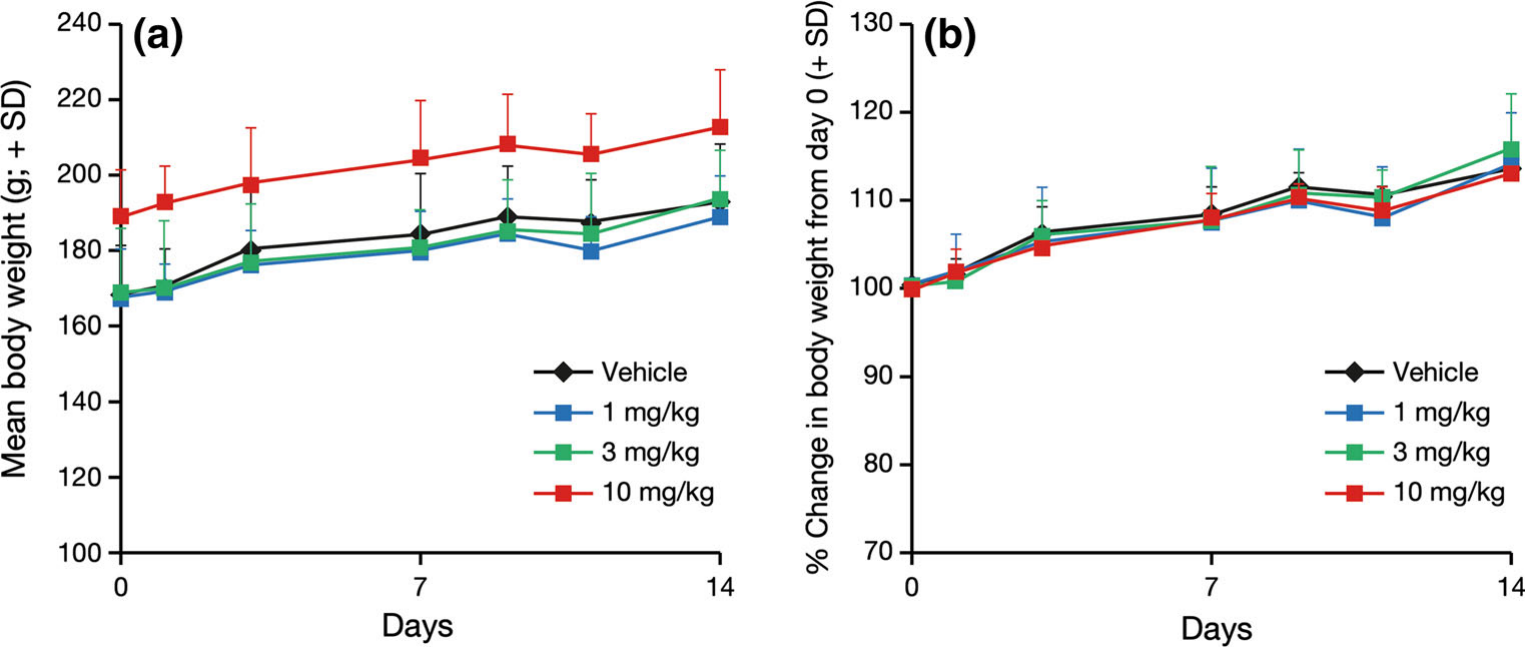
Effects of TAK-733 on the body weight of A549 tumor-bearing rats. A549 tumor-bearing rats were orally administered TAK-733 (1, 3, 10 mg/kg) or vehicle once daily for 2 weeks. Data are shown as mean + standard deviation (SD) (*n* = 8/group). *Black*, *blue*, *green* and *red* *lines* and *markers* indicate the vehicle, and TAK-733 1, 3, and 10 mg/kg dosage groups, respectively. *Left* body weight (g), *right* percentage change in body weight from day 0

TAK-733 was tolerated, and did not result in mean body weight loss at any dosage level (Fig. 2). There were no treatment-related deaths in any of the animals that received TAK-733 either.

**Fig. 3:**
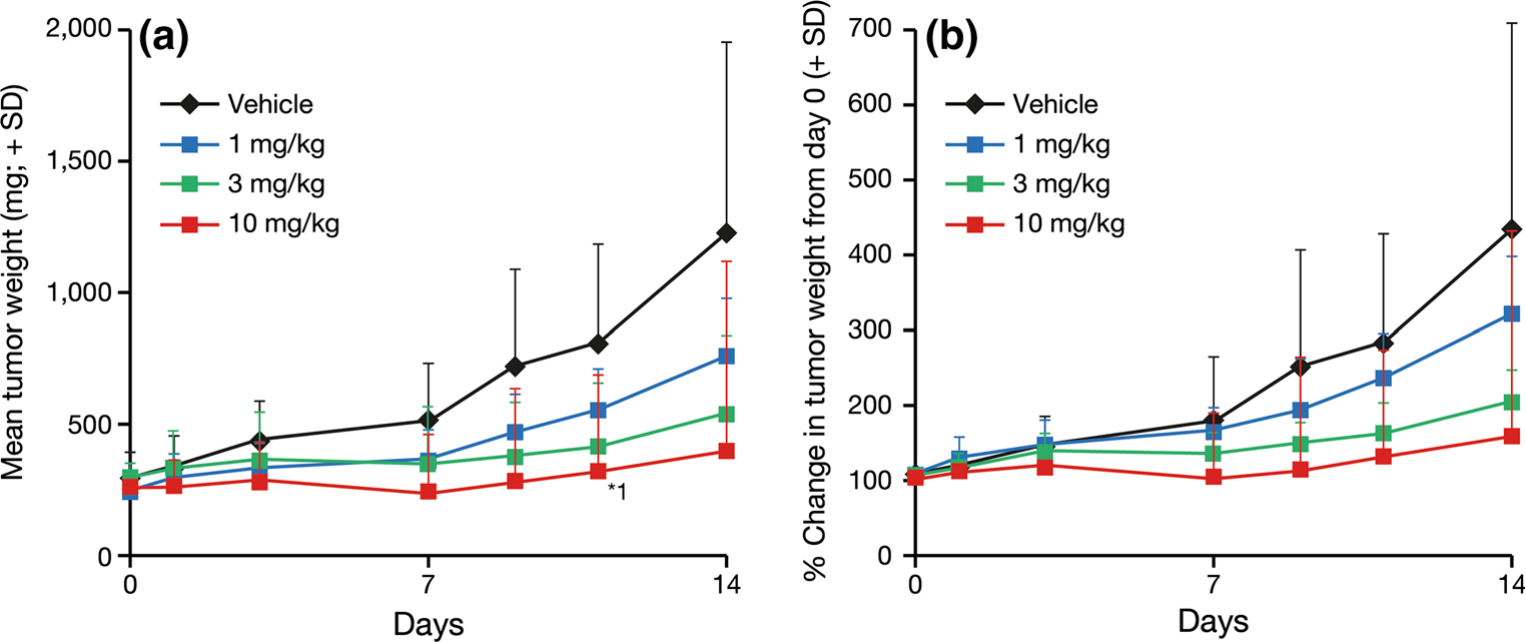
Effects of TAK-733 on the growth of human lung carcinoma A549 tumors in a xenograft rat model. Oral once-daily administration of TAK-733 (1, 3, 10 mg/kg) or vehicle was initiated when tumors reached a weight of 224–297 mg in A549 tumor-bearing rats. Data are expressed as mean + standard deviation (SD) (*n* = 8/group). *Black*, *blue*, *green* and *red* *lines* and *markers* indicate the vehicle, and TAK-733 1, 3, and 10 mg/kg dosage groups, respectively. *Left* A549 tumor weight (mg), *right* percentage change in tumor weight from day 0. *1: *P* < 0.05 vs. vehicle (Wilcoxon rank-sum test)

TAK-733 treatment resulted in dose-dependent tumor growth inhibition. TAK-733 at a dosage level of 10 mg/kg produced a statistically significant reduction in estimated tumor weight compared with vehicle administration on day 11 (mean + standard deviation, 314 + 161 vs. 817 + 375 mg, respectively; *P* < 0.05) (Fig. 3).

When dosed at 10 mg/kg, TAK-733 produced a minimum *T*/*C* value of 31 % on day 14, which is indicative of clinically significant anti-tumor activity based on the NCI definition (≤41 %) (Table 1). At 1 and 3 mg/kg, TAK-733 did not produce clinically significant *T*/*C* values on day 14 (55 and 44.0 %, respectively). There were no marked differences in *T*/*C* values among the three TAK-733 dosage groups during the early treatment phase (days 1 and 3), but there was a trend for dose dependency of *T*/*C* values during the later treatment phase (days 7, 9, 11, and 14) (Table 1).

**Table 1:** Mean %*T*/*C* values for median tumor weight by dosage group and day of study in A549 tumor-bearing rats receiving once-daily oral administration of TAK-733 (1, 3, 10 mg/kg) or vehicle for 2 weeks (*n* = 8/group)

TAK-733 dosage group	%*T*/*C* values by day of study						
	0	1	3	7	9	11	14
Vehicle	100	100	100	100	100	100	100
1 mg/kg	76	82	71	78	68	60	55
3 mg/kg	93	102	70	68	53	42	44
10 mg/kg	84	83	62	47	37*	36*	31*

%*T*/*C* ratio of median tumor weight in the treated versus control (vehicle) group × 100

*%*T*/*C* value ≤41 % (National Cancer Institute threshold for significant anti-cancer activity)

**Fig. 4:**
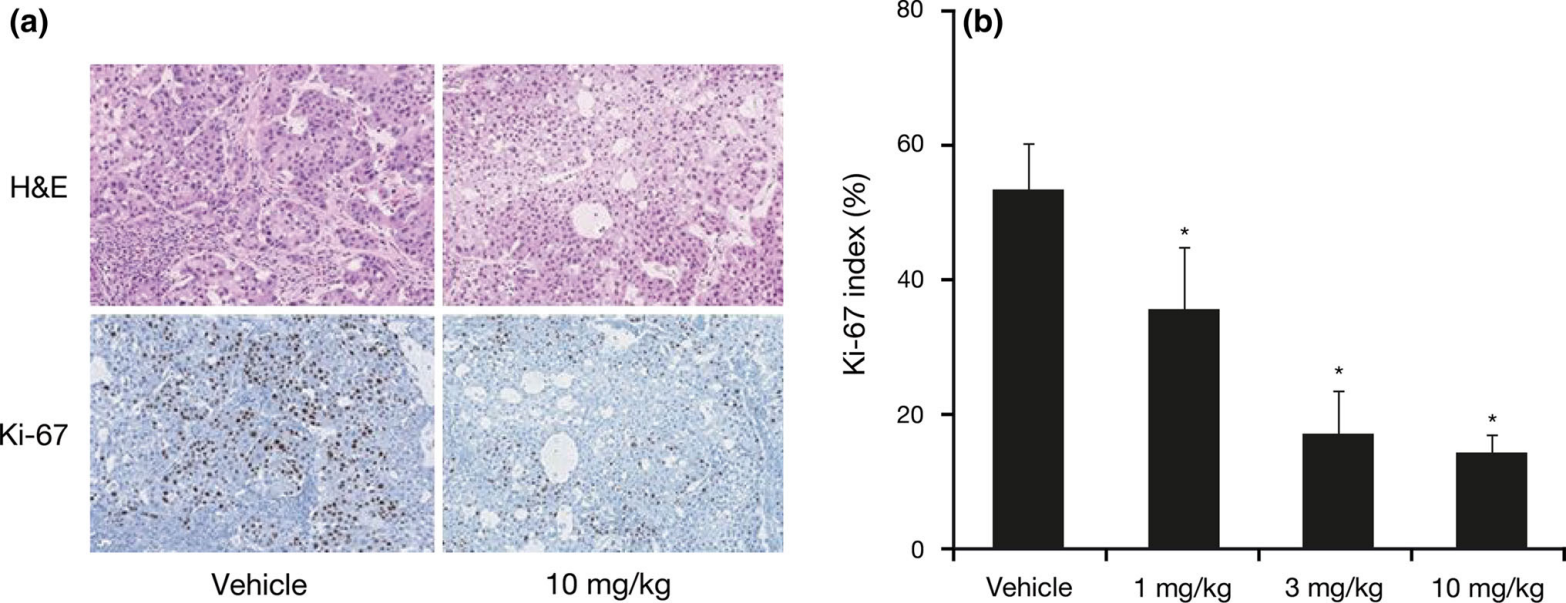
Anti-proliferative effects of TAK-733 in tumor tissue. **a** Representative images showing H&E staining and Ki-67 immunohistochemical staining in the vehicle and TAK-733 10 mg/kg dosage groups. Magnification is ×45. **b** Mean Ki-67 index in each dosage group (%). Data are expressed as mean + standard deviation (SD) (*n* = 8/group). *P < 0.025 vs. vehicle (Shirley–Williams test)

H&E staining images revealed tumor cell necrosis in the 10 mg/kg group (Fig. 4a). Staining showed pyknotic nuclei and eosinophilic cytoplasm in multifocal lesions. Ki-67-positive cells were frequently detected in the tumor tissue from the vehicle group; however, Ki-67 nuclear staining was barely detectable in the TAK-733 10 mg/kg dosage group (Fig. 4a). A statistically significant decrease in the Ki-67 index was observed in the 1, 3, and 10 mg/kg dosage groups (*P* < 0.025 vs. vehicle for all comparisons) (Fig. 4b).

### PET study

**Fig. 5:**
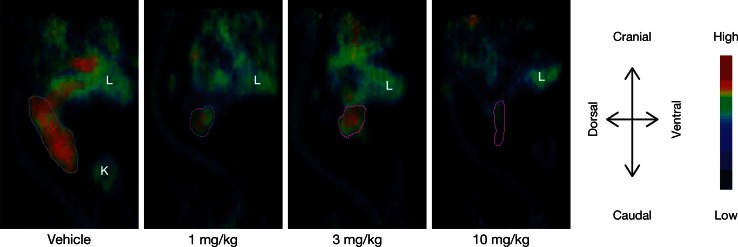
Representative ^18^F-FDG-PET images on day 14 following oral administration of TAK-733 (1, 3, 10 mg/kg) or vehicle once daily for 2 weeks in A549 tumor-bearing rats. The *purple line* border of implanted tumor ROI, the volume of which was consistent with caliper-based volume (average differences 1.08 %). *L* liver, *K* kidney

^18^F-FDG-PET images showed clear uptake of the tracer in tumor tissue, compared with background tissue in most cases. Relatively homogenous tumor uptake was seen throughout the study with only a few exceptions, where heterogeneous uptake was observed (Fig. 5).

**Fig. 6:**
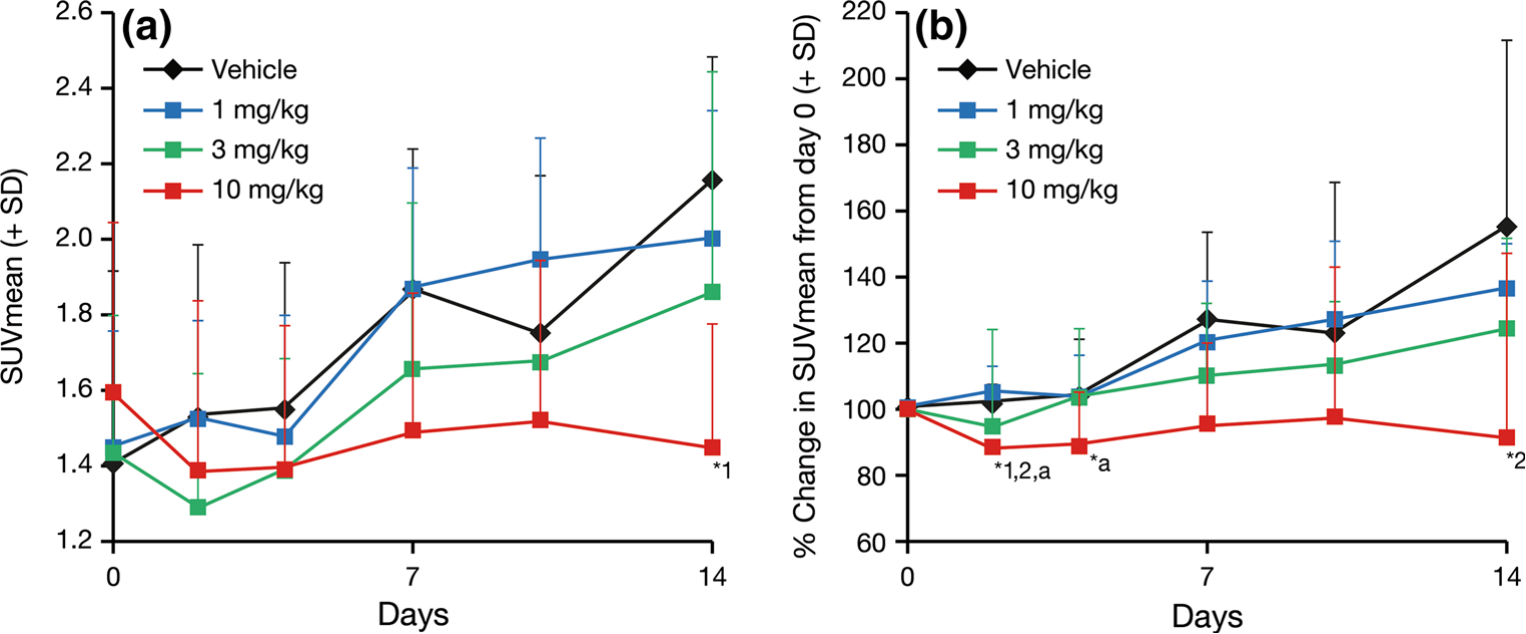
SUVmean values following TAK-733 (1, 3, 10 mg/kg) or vehicle treatment in human lung carcinoma A549 tumors in a xenograft rat model. ^18^F-FDG-PET scans were performed on days 0, 2, 4, 7, 10, and 14. Data are expressed as mean + standard deviation (SD) (*n* = 8/group). *Black*, *blue*, *green* and *red* *lines* and *markers* indicate vehicle, 1, 3, and 10 mg/kg group, respectively. ^*18*^ *F*-*FDG*-*PET* ^18^F-Fluorodeoxyglucose-positron emission tomography, *SUVmean* mean standard uptake. *Left* absolute SUVmean, *right* percentage change in SUVmean from day 0. *1: *P* < 0.05 vs. vehicle (Wilcoxon rank-sum test), *2: *P* < 0.05 vs. TAK-733 1 mg/kg (Wilcoxon rank-sum test), *a: *P* < 0.05 vs. pretreatment (Wilcoxon rank-sum test)

^18^F-FDG SUVmean gradually increased over time, and to a similar extent, in the vehicle and TAK-733 1 mg/kg groups (Fig. 6). A slight reduction in SUVmean was observed in the 3 mg/kg dosage group on day 2, but this change was not statistically significant (not significant change between: vehicle and 3 mg/kg dosage group and between pretreatment and mg/kg dosage group) and values increased gradually over time thereafter. In contrast, a 13.2 % reduction in SUVmean compared with pretreatment was observed in the 10 mg/kg group on day 2 (*P* < 0.05), which persisted throughout the study. The percentage reduction in SUVmean from day 0 at 10 mg/kg on day 2 was significantly different to that seen in the vehicle and 1 mg/kg groups (mean + standard deviation, 87 + 8 vs. 101 + 2 % and 105 + 8 %, respectively; *P* < 0.05 for both comparisons). On day 14, the absolute SUVmean at 10 mg/kg was significantly lower than that reported for the vehicle group (1.5 + 0.3 vs. 2.2 + 0.3, respectively; *P* < 0.05). Further, the percentage change in SUVmean at 10 mg/kg on day 14 was significantly different to that observed at 1 mg/kg (91 + 14 vs. 136 + 7 %, respectively; *P* < 0.05) (Fig. 6).

## Discussion

This preclinical study examined the correlation between early ^18^F-FDG-PET changes and changes in tumor size. The specific aims of the study were to evaluate whether TAK-733 treatment in rats bearing A549 human NSCLC xenografts resulted in an ^18^F-FDG-PET change, and whether the PET response change occurred earlier than morphological changes. Using this xenograft model of human lung carcinoma, we show that the anti-tumor effects of TAK-733 can be detected by ^18^F-FDG-PET.

In the TAK-733 10 mg/kg dosage group, the percentage change in ^18^F-FDG SUVmean on day 2 was statistically lower than that observed at pretreatment (*P* < 0.05), and statistically lower than the SUVmean in the vehicle and 1 mg/kg groups (both *P* < 0.05). In contrast, the SUVmean in the vehicle group, and TAK-733 1 and 3 mg/kg groups actually increased over time. On day 14, the SUVmean at 10 mg/kg (absolute value and percentage change from day 0) was significantly lower than that in the vehicle group (*P* < 0.05). TAK-733 is a novel allosteric inhibitor of MEK1/2, which not only impairs cell proliferation, but also impacts a diverse array of cellular events, including differentiation, apoptosis, and angiogenesis [1–4]. While ^18^F-FDG-PET can be used to detect tissues with abnormal glucose metabolism that may change in response to therapy, it is not yet clear whether TAK-733 itself directly affects glucose metabolic activity. A direct effect of a similar MEK inhibitor drug on glucose metabolism has been reported recently [15]; however, impairing cell proliferation and other cellular events will also alter glucose consumption levels in cancer cells. In the present study, the anti-proliferative effect was shown as Ki-67 index suppression on day 14 especially in the 10 mg/kg group with low ^18^F-FDG uptake, which indicates that the glucose consumption was inhibited via an anti-proliferative effect of TAK-733.

Compared with vehicle administration, treatment with TAK-733 10 mg/kg resulted in a statistically significant reduction in tumor weight on day 11, and a statistically significant decrease in the percentage change in ^18^F-FDG SUVmean from baseline on day 2. This statistical difference between the 10 mg/kg and vehicle groups did not persist, however, and was only observed at these single timepoints for both of these endpoints. As the PET response change (SUVmean, day 2) was observed earlier than the morphological changes (day 11), ^18^F-FDG-PET may have the potential to detect early responses to TAK-733 in lung carcinoma. Given the transient reduction in SUVmean at 3 mg/kg, it would be interesting to repeat these experiments using intermediate doses of TAK-733 (5 and 7 mg/kg) to fully evaluate the effect of treatment on ^18^F-FDG uptake.

As well as a marker of glucose metabolism, ^18^F-FDG uptake in tumors is also a marker of overall tumor energy consumption, which relates to the number of viable tumor cells [17, 18]. Reduction of glucose metabolism could represent a central indicator of overall anti-tumor activity, with various mechanisms leading to tumor cell death [19, 20]. These effects imaged by ^18^F-FDG-PET have been used as a pharmacodynamic marker of anti-tumor activity for a broad spectrum of anti-cancer therapies in clinical settings [16, 21–23]. Furthermore, recent studies in tumor models demonstrate that the reduction in ^18^F-FDG uptake also reflects the anti-proliferative effects of drug therapies and treatment-induced tumor apoptosis [24, 25]. In the present study, a dose-dependent ^18^F-FDG uptake regression was observed along with tumor size reduction (Figs. 3, 6) and anti-proliferative effect of TAK-733 was confirmed in the Ki-67 staining (Fig. 4). Therefore, we concluded that this ^18^F-FDG uptake regression can be used to predict tumor growth inhibition via the anti-proliferative effect of TAK-733. The PET tracer, ^18^F-FLT (3′-deoxy-3′-^18^F-fluorothymidine), can be used to detect cell proliferation. In consideration with the MEK inhibition pathway of TAK-733, Leyton et al. reported that ^18^F-FLT-PET is a sensitive imaging biomarker for detecting the anti-proliferative effect of MEK1/2 inhibition by PD0325901 (MEK inhibitor) [26]. However, as ^18^F-FDG-PET is widely used for cancer diagnosis, staging and treatment response, and is feasible for use in the clinical setting in comparison with ^18^F-FLT-PET, ^18^F-FDG-PET was selected as the preferred translational imaging modality for this study.

## Conclusion

Early response to anti-proliferative treatment in A549 lung carcinoma can be visualized with ^18^F-FDG-PET, and those changes in tracer accumulation after the treatment correlate with TAK-733 efficacy. Thus, ^18^F-FDG-PET has the potential to detect early responses to TAK-733 in lung carcinoma.
